# Unveiling the underlying drivers of Phanerozoic marine diversification

**DOI:** 10.1098/rspb.2024.0165

**Published:** 2024-06-19

**Authors:** Connor J. Wilson, Trond Reitan, Lee Hsiang Liow

**Affiliations:** ^1^ Natural History Museum, University of Oslo, 0562 Oslo, Norway; ^2^ Centre for Planetary Habitability, Department of Geosciences, University of Oslo, 0562 Oslo, Norway; ^3^ School of Geography and the Environment, University of Oxford, Oxford OX1 3QY, UK; ^4^ Department of Ecology and Evolutionary Biology, University of Arizona, Tucson, AZ 85719, USA

**Keywords:** origination, extinction, diversification, marine invertebrates, time series analysis, Granger causality

## Abstract

In investigating global patterns of biodiversity through deep time, many large-scale drivers of diversification have been proposed, both biotic and abiotic. However, few robust conclusions about these hypothesized effectors or their roles have been drawn. Here, we use a linear stochastic differential equation (SDE) framework to test for the presence of underlying drivers of diversification patterns before examining specific hypothesized drivers. Using a global dataset of observations of skeletonized marine fossils, we infer origination, extinction and sampling rates (collectively called fossil time series) throughout the Phanerozoic using a capture–mark–recapture approach. Using linear SDEs, we then compare models including and excluding hidden (i.e. unmeasured) drivers of these fossil time series. We find evidence of large-scale underlying drivers of marine Phanerozoic diversification rates and present quantitative characterizations of these. We then test whether changing global temperature, sea-level, marine sediment area or continental fragmentation could act as drivers of the fossil time series. We show that it is unlikely any of these four abiotic factors are the hidden drivers we identified, though there is evidence for correlative links between sediment area and origination/extinction rates. Our characterization of the hidden drivers of Phanerozoic diversification and sampling will aid in the search for their ultimate identities.

## 1. Introduction

Identifying the processes responsible for changes in biodiversity over time is one of the primary goals of palaeontologists and evolutionary biologists. Sepkoski [1,2] was one of the pioneers in examining how global patterns of biodiversity changed through deep time, and since then, increasingly sophisticated analyses of pattern and process have been performed. One particular area of focus has been to identify large-scale Earth system variables that have influenced biodiversity across broad taxa at large spatial extents and over long timescales. Many abiotic drivers have been proposed to have affected global patterns of biodiversity across the Phanerozoic, including temperature [3], sea-level [4], available habitat area [5] and the arrangement of the continents [6]. However, the results of many studies investigating these factors have been mixed and it is to date unclear if these proposed drivers are even necessary to explain large-scale patterns of biodiversity through the Phanerozoic, or if biotic factors such as diversity-dependence have broader explanatory power [7,8].

Changes in these large-scale abiotic drivers have been linked to patterns of diversity and diversification in numerous ways, including through empirical analyses of time series [9,10] and mechanistic simulations [11,12]. In this contribution, we take a ‘reverse’ approach to identifying drivers of diversification. Instead of starting out by comparing existing purported ‘driver’ time series data with diversification time series, we first investigate the possibility of the presence of underlying drivers, whatever their identities may be, that may be impacting temporal patterns of biodiversity. To do so we use a linear stochastic differential equation (SDE) framework that allows the characterization of unobserved drivers' effects on observed time series data [13]. The use of an SDE framework is a crucial improvement for inferring driving forces in the past [14,15]. This is because correlational or ordinary linear regression analyses, commonly applied in the palaeontological literature, not only are problematic for time series data *per se*, but also cannot distinguish between correlation and (Granger) causality (see review in [16]). Here, a time series representing an unobserved driver (one that is a Granger cause of the observed process) is termed a ‘hidden layer’, following [13,14]. If no hidden layers are found, there may not be observable variables that could be detected as drivers of diversification at the chosen scale of analysis. Alternatively, but less plausibly, a lack of detected hidden drivers could result from the process mimicking the driver so well that the two cannot be separated.

We test the plausibility of underlying, hidden drivers of marine biodiversity throughout the Phanerozoic using SDEs and characterize their structures. To do so, we first demonstrate that the linear SDE framework is able to infer hidden (unmeasured) layers from measured (observed) time series data using simulations. Then we estimate origination, extinction and sampling rates (henceforth 'fossil time series') of skeletonized marine invertebrates using capture–mark–recapture (CMR) approaches. Next, we characterize these three fossil time series and test for the presence of underlying structure using hidden layer SDE models. We find that hidden layers impact all three of our fossil time series and characterize each of these layers. Last, we proceed to evaluate the possibility that changing global temperature, sea-level, the availability of marine area, and continental fragmentation (henceforth 'abiotic time series'), commonly proposed effectors of biodiversity patterns, acted as drivers of one or more of our fossil time series. Although we conclude that none of these four abiotic factors is one of the drivers we identified via the hidden layer SDE analyses, our characterization of the hidden drivers of Phanerozoic diversification and sampling could aid in the search for their ultimate identities.

## 2. Methods

### (a) Diversification rate estimates

Observations of marine fossil taxa were downloaded from the Paleobiology Database (PBDB) (https://paleobiodb.org/) on 17 February 2022 without any temporal or geographical filters. Data downloaded were limited to observations of Brachiopoda, Bivalvia, Anthozoa, Trilobita, Gastropoda, Crinoidea, Blastoidea, Edrioasteroidea, Ammonoidea, Nautiloidea and Bryozoa that were identified to at least genus-level. Exact PBDB data output and the query used to generate it can be found in the electronic supplementary material. For estimation of fossil time series of origination, extinction and sampling rates, we used the Pradel seniority model (henceforth Pradel model) [17], a CMR open-population model that integrates forward and reverse time directions to estimate genus survival (the probability of persisting to the next time interval) and genus seniority (the probability of appearing in a previous time interval). Because the Pradel seniority model has been used in many different contexts in the palaeontological literature [18–21], we do not redescribe it, but emphasize that it uses information from the non-detection of taxa in time intervals where they must have survived (as they are observed again in subsequent time intervals), as well as those where the status of the taxon is ambiguous (non-detection due to non-existence, extinction, or the lack of sampling), in a modelling framework. The complements of survival and seniority are extinction and origination probabilities, respectively [19]. Alongside these variables, a fully time-varying Pradel model simultaneously estimates sampling probabilities within time intervals. We used the *openCR* [22] package in the R computing environment [23] to implement the CMR analysis. A capture history was created for each observed genus across the Phanerozoic stages [24,25]. This was then used to estimate time-varying origination and extinction probabilities across stage boundaries, and sampling probabilities within stages. To remove statistical edge effects, we discarded the first origination probability, last extinction probability, and the first and last sampling probabilities (those in the Fortunian and Holocene, respectively) [19]. The remaining probabilities were transformed into rates with respect to stage length using a Poisson model [18]. The code and data for the CMR analysis and all other analyses can be found in an Open Science Framework repository [26].

### (b) Hidden driver inference: a brief overview of linear stochastic differential equations

**Figure 1.**
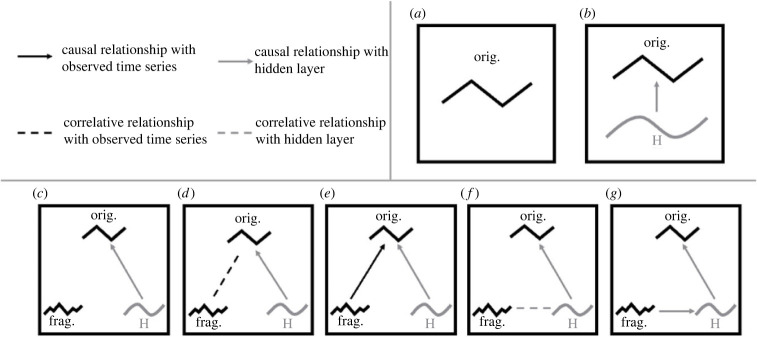
Models of how measured and hidden time series may be associated with one another, using origination and fragmentation as an illustration. At the top left is a key showing the different types of link models used to model relationships between time series. The top right shows the two classes of models we fitted to fossil time series to understand their underlying structure, where (*a*) shows a time series (using origination rates as an example) modelled as a single-layered process (i.e. OU or random walk) and (*b*) shows that time series modelled as a two-layered process (see table 1 for detail of the six models in this class). The bottom row shows the five models we fit to the fossil time series (again using origination rates as an example) after we have ascertained that it could have an underlying driver, which we hypothesized could be fragmentation, as an illustration. ‘H’ is the hidden layer, which we already ascertained is plausible. (*c*–*g*) Models including fragmentation in link analyses, where it had (*c*) no link to origination rates, (*d*) a correlative link, (*e*) a causal link, (*f*) a correlative link through a hidden layer, and (*g*) a causal link through a hidden layer.

To characterize our time series data and to investigate the possibility of hidden layers (unobserved underlying drivers) in the fossil time series, we use a linear stochastic differential equation (SDE) approach, the technical details of which are summarized in the electronic supplementary material and also in several earlier publications [13,14,18]. Linear SDEs have been used to investigate the relationships between bivalve and brachiopod diversification rates [18], cheilostome and cyclostome bryozoan diversification rates [27], coccolithophore size evolution [14], and other phenotypic time series. Briefly, an SDE is a continuous-time process expressed as a differential equation with at least one stochastic term [28]. Here, a process is a temporally changing quantity that can have varying degrees of predictability, an example of which is global temperature over the Phanerozoic. Analyses are usually couched in a specific linear SDE form, such as the Ornstein–Uhlenbeck (OU) process [29,30]. The OU process, widely used in evolutionary biology [30,31], has a deterministic term and a stochastic one. The deterministic term has parameters describing the mean (expected value) and characteristic time of the process, while the stochastic term describes variability (see sections below). Note that the mean in the deterministic term does not have to be constant in time (though it is in the OU process). To model two or more time series that are hypothesized to be related to each other, an SDE can be linked to other SDEs, with the connection characterized using coefficients determining link strength [14,18]. Links between two measured or observed time series can be modelled as correlations (via the stochastic term) or Granger causal relationships (where the ‘driver’ time series is ‘embedded’ in the deterministic term of the ‘driven’ time series, i.e. the mean of the driven series is ‘controlled’ by the ‘driver’ [32]). Granger causality [32] is a definition of causality that rests on information-transfer. Time series *X* is said to Granger cause time series *Y* if the present state of *Y* can better be predicted by the past of both *X* and *Y* than by only the past of *Y*. Such links can also be estimated between a measured and an unmeasured time series (i.e. a hidden layer, figure 1). We use an SDE approach for its capabilities described above, but also because it offers other advantages. For example, this method is amenable to unevenly spaced time points (hence ad hoc temporal binning is unnecessary) and less prone to false inferences than approaches like first differencing [16].

**Table 1.** Comparisons of different models of fossil time series. This table summarizes the results of hidden layer analyses of the three fossil time series. For each time series, a hidden layer model, i.e. a model with a potential driver, was the best (shown in bold type under corrected Akaike information criterion ('AICc') and 'model weight').

model	origination		extinction		sampling	
	AICc	model weight	AICc	model weight	AICc	model weight
1 (1-OU)	224.11	0.00019	264.35	0.33	168.69	0.026
2 (1-RW)	274.59	0.00	336.64	0.00	193.59	0.0000001
3 (2-OU)	**207.65**	**0.70**	270.20	0.018	**162.61**	**0.55**
4 (2-loop)	209.42	0.29	271.70	0.0085	164.66	0.20
5 (2-OU-det)	223.31	0.00028	268.35	0.045	173.71	0.0021
6 (2-OU-det-2-way)	225.21	0.00011	270.35	0.017	174.85	0.0012
7 (2-OU-RW)	216.96	0.0067	**263.26**	**0.58**	164.42	0.22
8 (2-OU-RW-det)	278.53	0.00	340.0030	0.00	196.46	0.00

### (c) Hidden driver inference: a demonstrative simulation

It may seem unintuitive that drivers that are not directly observed or measured can be detected at all. Hidden layers can be detected because they leave an imprint on the correlation structure beyond what can be expected in a single linear SDE (electronic supplementary material, figure S3; [14,15] for technical details). Here, we supply the code for simulating a system where an underlying layer drives a ‘top layer’. We then apply the linear SDE framework via the R package *layeranalyzer* [15] to the ‘top layer’ only (without the simulated underlying layer, i.e. it becomes ‘hidden’ to us), to demonstrate that the underlying layer can be inferred.

### (d) Hidden driver analyses (empirical analyses)

We first test for the presence of hidden layers in each of our three fossil time series (see previous section) separately by comparing eight models that either only have single layers, i.e. no hidden layers and hence no opportunity for detecting any underlying empirical drivers (figure 1*a*) or have two layers, one of which is hidden, hence a potential for identifying underlying empirical drivers (figure 1*b*). These are termed ‘standalone’ analyses. Model 1 (1-OU) is a single-layered model where the given time series is modelled as an OU process with an expected value (*μ*), a characteristic time (d*t*) and a standard deviation (*σ*). Model 2 (1-RW) is a single-layered model where the time series is modelled as a random walk with expected value (*μ*) and standard deviation (*σ*). Models 1 and 2 hence belong to the class of models where no underlying drivers are incorporated (figure 1*a*). Model 3 (2-OU) is a two-layered model where the hidden layer is an OU process and the top layer is an OU-like stochastic tracking process that is influenced by the hidden layer (figure 1*b*). Model 3 could, for instance, be a case where fragmentation changes (hidden layer) drive extinction rate (top layer). Model 4 (2-loop) is a two-layered model where the two processes are OU-like but where they influence each other in a feedback loop. Model 4 could, for instance, be a case where global temperatures (hidden layer) drive origination rates (top layer), which in turn modify global temperature (e.g. via the release of more greenhouse gases through metabolism). Model 5 (2-OU-det) is like model 3 (2-OU) except that the observed process is deterministically tracking the process in the hidden layer, i.e. its response is completely deterministic. Model 6 (2-OU-det-2-way) is like model 5, except that the observed time series also influences the hidden layer. Model 7 (2-OU-RW) is a two-layered model where the observed time series is a stochastic, OU-like tracking layer responding to the unobserved hidden layer, which is a random walk. Lastly, model 8 (2-OU-RW-det) is like model 7 except that the observed time series is a deterministic tracking layer responding to the hidden layer (see electronic supplementary material for detailed descriptions of these models and an explanation of OU-like processes). We compare these eight models separately for each fossil time series, using the corrected Akaike information criterion (AICc) to assess which models (with or without hidden layers) are best supported. If hidden-layer models outperform single-layer ones, that would be evidence for underlying structure and drivers to one or more of the three fossil time series. As we do in fact find hidden layers (see Results), we extract and visualize the modelled hidden layers for each of the three fossil time series from the best hidden layer models (figure 4). In addition, we subtracted the hidden layer from the top layer to reveal the observed process (top layer) without the influence of the hidden layers in our time series (electronic supplementary material). We implement our SDE models using the R package *layeranalyzer* [15] after log-transforming each of the fossil time series for normality.

### (e) Testing four abiotic time series as underlying drivers of fossil time series

**Figure 2.**
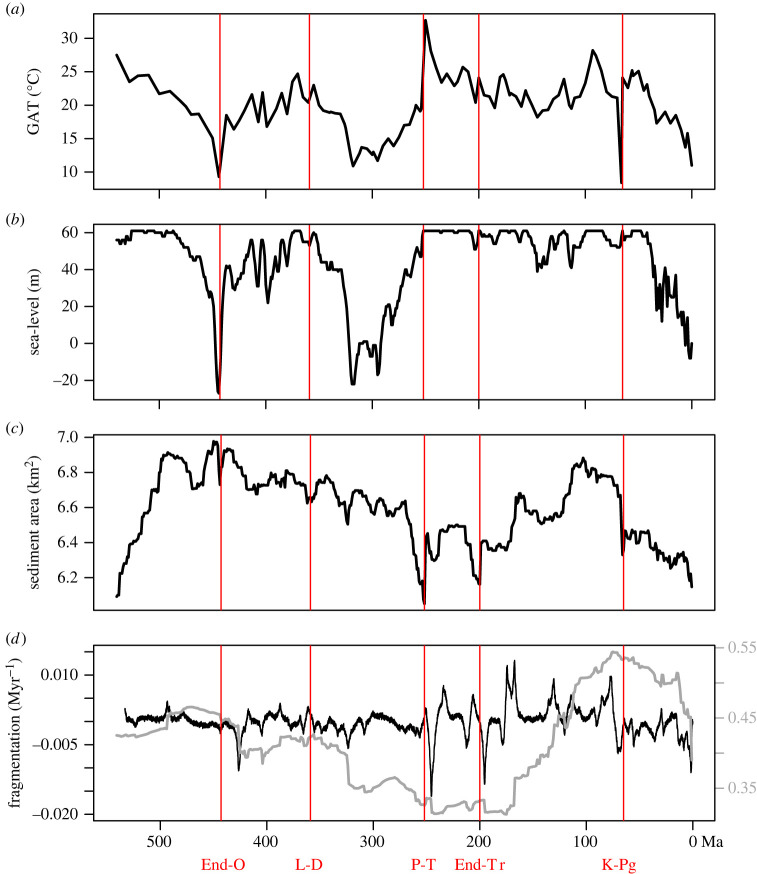
Abiotic time series input for linear stochastic differential equation (SDE) analyses. (*a*) Global average temperature (GAT, in °C) from [35]. (*b*) Sea-level (m) from [37]. (*c*) Marine sediment area (km^2^, on log_10_-scale) from [40]. (*d*) Fragmentation index (grey line and grey vertical axis on the right) from [9] and the estimated fragmentation rate (index change per million years) used in the hidden layer analyses. Vertical red lines show the timings of the ‘big 5' mass extinctions: the End Ordovician (End-O), Late Devonian (L-D), Permian-Triassic (P-T), End Triassic (End-Tr) and Cretaceous-Palaeogene (K-Pg) events.

We investigate four relatively newly published abiotic time series as potential drivers of the fossil time series (figure 2). They are global temperature, sea-level fluctuations, marine sediment area and continental fragmentation. Temperature change has long been hypothesized to be associated with changes in diversity, both empirically and theoretically [33,34]. However, results have been heterogeneous and analyses using different data and approaches often contradict one another [18,34]. We use the Phanerozoic global average temperature reconstruction given in [35] for our analyses. Sea-level fluctuations, suggested to reflect changes in the general state of the marine realm, have also been hypothesized as a potential driver of marine diversity, though their effects may be confounded/mediated through other Earth system variables [4,36]. We use a global sea-level reconstruction from [37], said to be a mixture of tectonic, eustatic and glacial signals. The relationship between diversity and habitat area is one of the most universal patterns in ecology [38], and the availability of marine habitat has been hypothesized to modulate biodiversity over deep time [5,39]. Here, we use log-transformed Phanerozoic marine sediment area data from [40] to represent habitat area, though it could also be related to other geological or taphonomic processes [41,42]. Likewise, continental fragmentation has been hypothesized to drive diversification throughout the Phanerozoic [6]. In this study, we use the fragmentation index [9], a proxy for continental fragmentation. Index values are at their lowest when all continental blocks are touching, with minimized coastlines, and at their highest when all continental blocks are separate. We use the rate of continental fragmentation as input because many standing biological hypotheses imply the dynamics of fragmentation, not just the amount of coastline, in driving diversification rates. To get the rate of fragmentation, we estimate the derivative of the normalized fragmentation index from [9] using the linear SDE framework, implemented in *layeranalyze*r, to minimize issues regarding the statistical non-independence of time points (see electronic supplementary material). We subsample the estimated derivative (i.e. fragmentation rate) to million-year intervals, the same resolution as the fragmentation index, and utilize it in our main SDE analysis (figure 2*d*). To test whether any of these four abiotic time series are related to diversification processes or sampling, we compare models in which each abiotic time series are unlinked with the fossil time series to those in which they are linked, either correlatively or causally, using classical hypothesis testing (see below). We call these link analyses.

**Figure 3.**
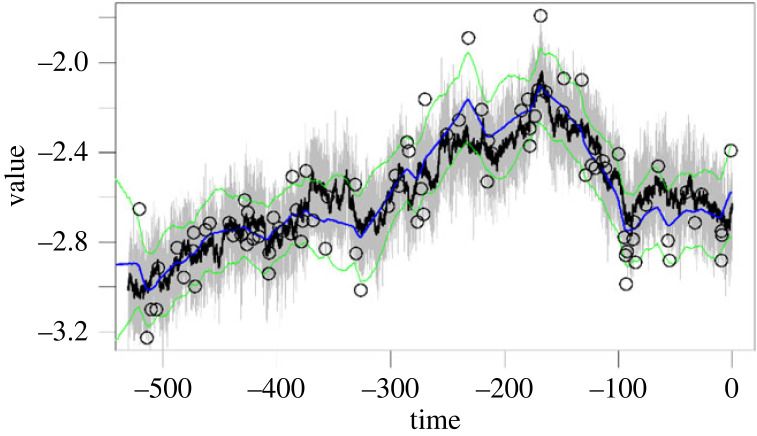
Inferring the hidden layer. This figure shows simulated data, the dynamics of which were generated by an underlying layer. The open circles are the observations (from the simulated ‘top layer’, grey solid line) that *layeranalyzer* used for inference (like the observations we have in our fossil and abiotic time series) while the black line is the underlying hidden layer that we do not ‘observe’. The blue line is the inferred hidden layer (mean) with its 95% confidence intervals (green). Note that the ‘observations’ (black circles) are really a combination of the black and grey processes; hence our ability to reconstruct both of them (even though only the hidden reconstructed layer is shown in blue and green).

**Figure 4.**
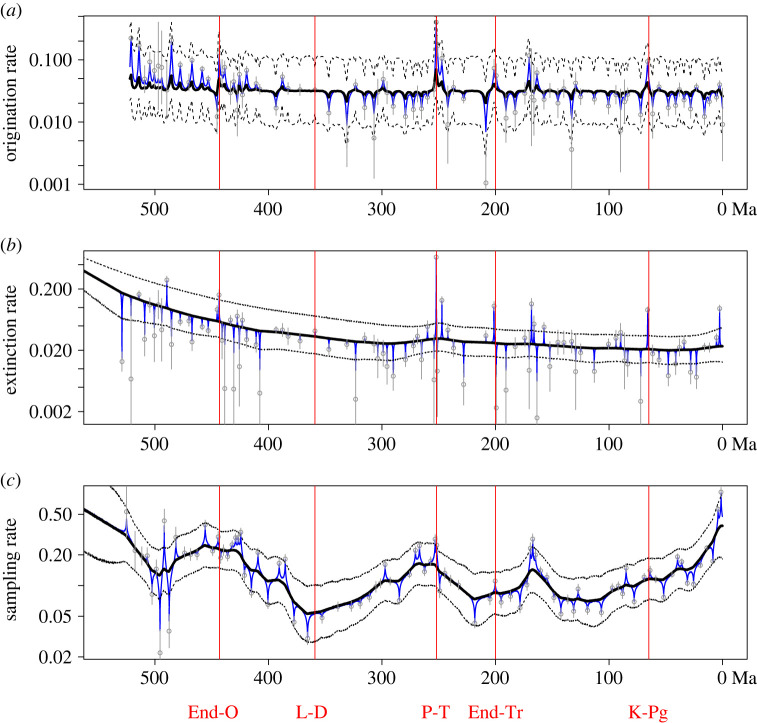
Estimated hidden layers underlying fossil time series. This figure shows the estimated hidden layers underlying time series of (*a*) origination rate, (*b*) extinction rate and (*c*) sampling rate. Bold black lines are the mean estimate of each hidden layer, and the dashed black lines represent upper and lower 95% confidence bounds for the hidden layer. The blue lines are the estimated top layers for each process. For visibility, the much greater uncertainties of the top layers are omitted from this figure (see electronic supplementary material for further detail). Original fossil time series data in grey. Vertical red lines show the timings of the ‘big 5’ mass extinctions: the End Ordovician (End-O), Late Devonian (L-D), Permian-Triassic (P-T), End Triassic (End-Tr) and Cretaceous-Palaeogene (K-Pg) events.

Each comparison involves an abiotic time series and a fossil time series. For each time series pair, five models are compared (letter labels from figure 1 correspond to the letters used to identify the models in table 2): (1) a null model with no links between the pair (figure 1*c*, illustrated using origination rates and its hidden layer), (2) a model in which the two observed time series are temporally correlated (figure 1*d*), (3) one in which they are linked by a Granger causal connection (figure 1*e*), (4) a model in which the abiotic time series (illustrated using fragmentation) is correlated with an underlying hidden layer driving rates estimated from fossil time series (figure 1*f*), and (5) a model in which the abiotic time series is Granger causally driving the hidden layer, which in turn is driving the rates estimated from fossil observations (figure 1*g*). In this instance, hidden layer models allow us to account for a scenario where there is a mediating process between the observed abiotic variable and diversification, such as rate of new habitat generation or spatial isolation with disruption of potential gene flow. There is one exception: sea-level itself has a hidden layer, such that there are extra link models, beyond those described above, to be considered (see Results). Here, we want to establish whether there are links between pairs of time series; hence we use classical hypothesis testing (against the relevant null model of no links in each case), with Bonferroni corrections to account for multiple testing, rather than AIC-based model selection, which would only tell us which model is best among those studied [43]. The total number of link model tests conducted is 54 (four link models for three fossil time series each for temperature, marine area and fragmentation, and six link models for sea-level because of its own hidden layers), required as input for the Bonferroni corrections to the ANOVA *p*-values when compared with null models.

## 3. Results

### (a) Diversification rate estimates

Origination and extinction rates across stage boundaries show fluctuations associated with the ‘big 5’ mass extinctions (figure 4*a,b*), and extinction shows a general decline over the Phanerozoic, while sampling rates show substantial variability throughout, as expected (figure 4*c*). It is notable that some time intervals have better constrained estimates of origination and extinction than others (figure 4). This variation in uncertainty is accounted for in downstream analyses of these fossil time series in the *layeranalyzer* framework. We also present the original fragmentation index from [9] alongside the estimated fragmentation rate from the linear SDE framework (figure 2*d*), as we derived it, unlike the other three abiotic time series. Note that the fragmentation index was not followed with error estimates in [9] and for consistency we supply only point estimates of fragmentation rates.

### (b) Inferring hidden layers

In figure 3, we show a simulation to illustrate the feasibility of inferring a hidden layer. While this is only a single rendition, our code is provided [26] for further demonstration.

### (c) Structure of the fossil time series: the presence of hidden layers

Our standalone analyses of time series of origination, extinction and sampling rates reveal the presence of hidden layers for all three processes. In our model comparisons, the best-fitting model for each of the three fossil time series contains a hidden layer explaining some of the variation of the process (table 1). Our analysis of origination rates reveals strong support for a model where origination rates follow an OU process that is driven by a different, underlying OU process (model 3, 2-OU with 70% model weight in table 1; see electronic supplementary material, table S2 for parameter estimates and figure 4*a* for the estimated processes). For extinction rates, the best model is also a two-layered model, but where the observed time series is driven by an underlying random walk process (model 7, 2-OU-RW, with 58% model weight in table 1; see also figure 3*b*). Sampling rates have the same structure as origination rates where the best is also a two-layered model (model 3, 2-OU, with 55% model weight in table 1; figure 4*c*) although the time scale at which the underlying layer acts is shorter than that of origination rates. The top layers of each time series are rapid OU-like processes, accounting for the short-term variation in the data (see electronic supplementary material, tables S2 and S3 for parameter estimates, including the characteristic times of the OU-like processes).

### (d) None of the four abiotic time series is a statistically significant driver of fossil time series

Our link analyses do not support any Granger causal connections between the four abiotic time series (parameter estimates from the standalone analyses are provided in the electronic supplementary material for completeness) and marine invertebrate diversification or sampling, nor to the hidden layer process found behind each observed process, given the Bonferroni correction (for 54 tests, table 2). However, marine sediment area has a significant correlative link to origination and extinction rates. Note that the correlative link implies that the marine sediment area and origination rates might have a common cause but does not imply that marine sediment area drove origination or extinction rates (see Discussion).

**Table 2.** Classical hypothesis tests for link analyses for fossil and abiotic time series. Each block corresponds to a set of tests for a particular abiotic variable and the set of fossil time series. *p*-Values are reported where the Bonferroni limit is 0.05/54 = 0.00093. The letters following the model names in the first column correspond to the letters used to identify the models from figure 1. Note that sea-level has a distinct set of models as it has a hidden layer.

	origination	extinction	sampling
*global temperature*			
correlative link to measured layer (*d*)	0.052	0.10	1
causal link to measured layer (*e*)	0.13	0.98	0.18
correlative link to hidden layer (*f*)	0.085	0.22	0.055
causal link to hidden layer (*g*)	0.16	0.98	0.28
*sea-level*			
correlative link from hidden layer to measured layer (*d*)	0.36	0.23	0.83
causal link from measured layer to measured layer (*e*)	0.14	0.82	0.35
causal link from hidden layer to measured layer (*e*)	0.11	0.84	0.21
correlative link from hidden layer to hidden layer (*f*)	0.017	0.21	0.13
causal link from measured layer to hidden layer (*g*)	0.20	0.74	0.41
causal link from hidden layer to hidden layer (*g*)	0.23	1	0.17
*marine sediment area*			
correlative link to measured layer (*d*)	0.14	0.000048	0.61
causal link to measured layer (*e*)	0.0048	0.22	0.18
correlative link to hidden layer (*f*)	0.00011	0.000014	0.15
causal link to hidden layer (*g*)	0.0099	0.32	0.41
*fragmentation rate*			
correlative link to measured layer (*d*)	0.19	0.70	0.70
causal link to measured layer (*e*)	0.99	0.91	0.71
correlative link to hidden layer (*f*)	0.31	0.67	1
causal link to hidden layer (*g*)	0.94	0.94	0.76

## 4. Discussion

The search for overarching patterns in the history of life's diversity and the desire to explain those patterns has been a prime motivator for evolutionary biologists and palaeontologists. The question of whether Phanerozoic diversification patterns are governed by consistent rules/drivers or are the result of random processes is a persistent problem [44,45]. To test this question, many specific putative drivers, such as competition/density-dependence [27,46], climate [3,34] and geography [6,9], have been investigated. By taking a ‘reverse’ approach, our analyses reveal that there are indeed long-term, taxonomically broad factors underlying marine Phanerozoic patterns of origination, extinction and sampling.

The high support given to the hidden layer models when compared with the single-layered ones indicates a high likelihood of underlying structures to the data, whether they are external drivers or internal patterns. We note that the hidden layers we uncover have much lower uncertainty than the top layers which are most proximate to the observed data. Owing to the speed and noisiness of the top layers (see also reconstructed top layers in the electronic supplementary material), it is also possible that the hidden layers represent the ‘true’ time series of origination, extinction or sampling, and the top layers represent unaccounted for measurement noise. Regardless, we demonstrate that structures underlying these time series exist and that these structures can be investigated. Furthermore, by extracting the hidden layers from our analysis, we provide a quantitative foundation for investigation of long-term drivers of diversification rates. Each of the underlying drivers of our fossil time series has distinct properties, suggesting possible differences in the natures of these processes. Potential drivers that display temporal variation similar to our uncovered hidden layers may be ideal candidates to test their relation to diversification. We note, however, the mathematical limitations of our hidden layer extraction. The estimated processes are without scale or sign, meaning that the processes they represent in the real world could be inverted or with a different absolute magnitude of data points.

Our results show little evidence for a causal connection between either global temperature (GAT), sea-level, marine sediment area or continental fragmentation rates and either macroevolutionary or fossil sampling rates of skeletonized marine invertebrates at a global scale when a process-model-based, formal time series approach (linear SDEs) is used. In contrast to our study, much of the published palaeontological literature uses relatively ‘naive’ methods to investigate proposed ‘drivers’ of evolutionary change, such as applying simple Pearson correlation or ordinary linear regressions on time series data, which do not account for temporal autocorrelation.

Temperature has been hypothesized to impact diversification in several ways, including altering speciation and extinction rates via changes in metabolism and energetics [33] and by mediating extinction caused by other abiotic variables such as oxygenation [11]. Thus far, empirical evidence has been equivocal, with studies finding conflicting results, possibly owing to the interactions of multiple factors and varying scales of analysis [18,34,47]. With all the models we fit having *p*-values above the Bonferroni limit, our results are consistent with GAT not playing a direct role in governing Phanerozoic diversification patterns on the timescales of our analyses (lowest *p*-value is *p* = 0.052 > 0.05/54, for a correlative link to a measured layer). Sea-level has been argued to be related to available marine habitat area and the overall geophysical state of the marine realm, though analyses at different temporal and spatial scales have found contrasting patterns [4,36]. The impact of other variables such as habitat type and geography may make the relationship between sea-level and habitat area indirect and idiosyncratic [36]. With all *p*-values above the Bonferroni limit (most of them substantially above), our analyses are consistent with the hypothesis that sea-level is not directly linked to the diversification history of marine invertebrates, just like in the case of GAT.

The relationship between sedimentary area and diversity has long been subject to investigation [42]. Hypothesized mechanisms by which sedimentary area may drive changes in observed diversification patterns include changes in habitable area, preservational biases or the dual forcing of both variables by a common driver. Although not supported as a causal underlying driver, marine sediment area has a correlative link to the hidden layer of the origination time series at *p* = 0.00011 and a correlative link to the measured and hidden layers of extinction at *p* < 0.00005. The statistical association between sediment area and origination rates is positive, while that between sediment area and extinction rates is negative. The correlational nature of the links between sediment area and origination/extinction suggests a possible common cause mechanism, where an Earth system driver impacts patterns of both sedimentary rock deposition and diversification. For example, the extent of shallow seas has been hypothesized to impact the accumulation of both biological diversity through species-area effects and sedimentary rock through area available for deposition [41]. Continental fragmentation has been hypothesized to impact biodiversity through various mechanisms, including biogeographical provinciality, habitat area, nutrient availability, and global climate [6,9,48]. Various investigations, including empirical time series analyses [9] and simulations [12,49], have found conflicting evidence for the role of fragmentation in controlling patterns of diversification. We find no detectable connection between continental fragmentation rates and diversification, utilizing more advanced time series tools than some past efforts [9]. Our results do not imply that these abiotic factors had no impact on the evolution of the biosphere, only that there are no detectable impacts on the timescales of the data available. The temporal and spatial composition of the biota could still have been affected by temperature, habitat availability and other physical conditions of their surroundings in other ways and on different timescales.

We find the abiotic time series and fossil time series in our SDE analyses to have varying characteristic times, meaning that variation and change within these processes happen on different timescales from one another. For example, temperature, sediment area and fragmentation rate have characteristic times on the order of tens of millions of years, while the fossil rates have characteristic times of around 1 million years or less (table 3). This timescale mismatch in part explains the lack of support for causal models. This result could imply a biological interpretation: some of these abiotic processes operate on a different timescale compared with origination and extinction and are therefore unlikely to play a large or detectable role in driving the latter processes. Using the hidden layer models, we check if intermediate processes could causally bridge the abiotic time series and the fossil time series, but this is not supported by the analyses.

**Table 3.** Characteristic times of modelled time series. This table contains the characteristic times for the most favoured models for each time series. The hidden layers for origination and sampling also contain a characteristic time parameter, which is included. Extinction and sea-level also have a hidden layer, but with a random walk (RW) process as the bottom layer (which can be interpreted as a characteristic time larger than the timespan of the data).

	origination	extinction	sampling	temperature	sea-level	area	fragmentation rate
characteristic time (Myr)	1.3	0.11	1.3	34	2.9	42	12
hidden layer characteristic time (Myr)	60	RW	41	NA	RW	NA	NA

Though we test only abiotic time series herein, biotic processes may have played a vital role in determining long-term patterns of diversification. Many analyses have found evidence of biotic processes impacting diversification, including equilibrial dynamics [50] and diversity-dependence of taxonomic rates [8,46]. It is also known that across many systems origination and extinction rates are closely tied [18,51]. We do not analyse the relation between origination and extinction in our study to avoid any issues with the time series being confounded owing to being generated from the same data. Despite this, testing the impacts of biodiversity upon itself using the SDE framework is an area ripe for future research.

Evidence for synthetic effects of multiple drivers, such as the impacts of geography and climate together, has been found [52], and could also be investigated over the Phanerozoic. Furthermore, biotic variables may interact with the abiotic ones we included; it is possible, for example, that the inferred underlying drivers represent the impacts of the interaction of biotic competition, ecological variation, and climatic variables [53]. The interaction of multiple drivers to produce diversification patterns is another area amenable to exploration with SDE methods.

We conduct our analyses at the level of genera, which may not have perfect congruence with biological dynamics at the species level [54]. However, a good proportion of morpho-genera are likely monophyletic [55], allowing the analysis of the diversification of both lineage and form, with the advantages of higher taxonomic consistency [56].

In this paper, we demonstrate the existence of large-scale underlying drivers of marine invertebrate origination, extinction and sampling during the Phanerozoic, signalling that the long-running search for broad structures governing diversification is not just a fool's errand. The drivers we identify may be internal or external to the biota and composed of single or multiple interacting variables. We tested four hypothesized drivers, and though there may be a correlational relationship with marine sediment area, the main source of the inferred underlying drivers remains unknown. Since our results do not support unequivocal causal connections between any of our tested drivers and diversification, we suggest further exploration of plausible variables.

## Data Availability

Code and data for all analyses can be found in an Open Science Framework repository: http://doi.org/10.17605/OSF.IO/5JAFU [26]. Additional information and results are provided in the electronic supplementary material [57].
